# Supplementary material for: Repeat modules and N-linked glycans define structure and antigenicity of a critical enterotoxigenic E. coli adhesin
Source: bioRxiv. 2024 May 8:2024.05.08.593125. Preprint. [Version 1] doi: 10.1101/2024.05.08.593125 (PMC11100705; doi:10.1101/2024.05.08.593125)
Supplement: Supplement 1 [file NIHPP2024.05.08.593125v1-supplement-1.pdf]

bioRxiv preprint doi: https://doi.org/10.1101/2024.05.08.593125; this version posted May 8, 2024. The copyright holder for this preprint (which was not certified by peer review) is the author/funder. This article is a US Government work. It is not subject to copyright under 17 USC 105 and is also made available for use under a CC0 license.

Supplemental information

Statistical analysis of EtpA N-linked glycosylation

Considering the extreme heterogeneity in occupancy and the relaxed sequence preferences of the EtpC glycosyltrasferase as revealed by MS, we sought to determine if there were any statistically significant trends in the frequency of amino acid types flanking PNGS that could potentially explain this data. To do this, we calculated the frequencies of each amino acid type at the two sequence positions upstream and downstream of each glycosylated PNGS for both sequon and non-sequon sites binned into either the top or bottom half of occupancy percentage as well as those sites that did not harbor any glycan modifications (0% occupancy) (Supplemental Figure 5). Looking at sequon PNGS only (Supplemental Figure 5A), we see potential trends emerge. First, there is a ~3:1 bias for T over S at the N+2 position (relative to the glycosylated Asn) in all occupancy bins as well as a significant enrichment T in the N-2 position, the latter of which could be explained by the common occurrence of multiple PNGS in a row. Other trends include an enrichment of isoleucine (I) at the N+2 position among glycosylated PNGS as well as S at the N-1 position and alanine (A) at the N-2 and N+1 position among the highest occupancy sites. 7 out of the 8 sequon PNGS with 0% occupancy occur at the same sequence/structural motif, namely, at the second to last residue on the β-strand of PB3 (Supplemental Figure 5B). Looking at non-sequon PNGS, one potential trend is the enrichment of the small non-polar residues glycine (G) and A at several of the flanking sites (Supplemental Figure 5C). Other residues that are enriched around non-sequon sites

bioRxiv preprint doi: https://doi.org/10.1101/2024.05.08.593125; this version posted May 8, 2024. The copyright holder for this preprint (which was not certified by peer review) is the author/funder. This article is a US Government work. It is not subject to copyright under 17 USC 105 and is also made available for use under a CC0 license.

harboring glycans are I, S, and T. Notably, there are only 13 non-sequon PNGS that have >=50% occupancy representing 3 unique sequence motifs that almost all occur at the same structural motif, namely, at the first residue of a β-strand immediately after a loop separating faces of the β-helix (Supplemental Figure 5D).

bioRxiv preprint doi: https://doi.org/10.1101/2024.05.08.593125; this version posted May 8, 2024. The copyright holder for this preprint (which was not certified by peer review) is the author/funder. This article is a US Government work. It is not subject to copyright under 17 USC 105 and is also made available for use under a CC0 license.

Supplemental figures

Supplemental figure 1

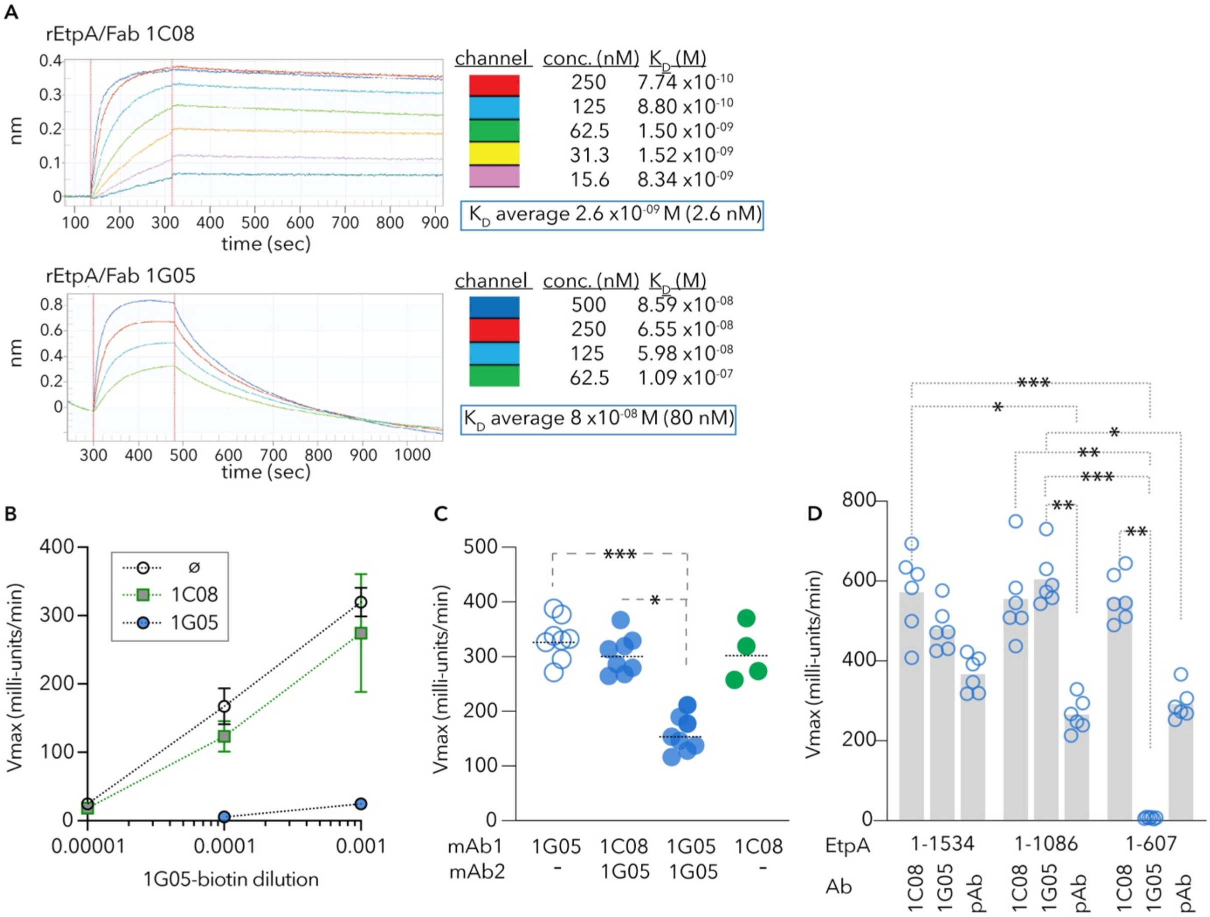

Supplemental figure 1. Anti-EtpA mAbs exhibit distinct affinities to unique regions of the antigen. A. Biolayer inferometry (Octet) studies 1C08 and 1G05 Fabs binding to rEtpA. **B.** mAb 1C08 does not compete for binding with 1G05. Shown are kinetic ELISA data indicating binding of bioinylated 1G05 mAb in the presence of unlabeled 1G05 (blue), 1C08 (green) or alone (open circle). **C.** 1G05 and 1C08 recognize EtpA but compete for different binding

bioRxiv preprint doi: https://doi.org/10.1101/2024.05.08.593125; this version posted May 8, 2024. The copyright holder for this preprint (which was not certified by peer review) is the author/funder. This article is a US Government work. It is not subject to copyright under 17 USC 105 and is also made available for use under a CC0 license.

sites. **D.** 1G05 recognizes the repeat region of EtpA while 1C08 binds the N-terminal secretion domain. Data include n=6 technical replicates and are representative of three independent experiments. pAb=polyclonal anti-EtpA antibody. Comparisons by Kruskal-Wallis ***≤0.001, **≤0.01, *<0.05.

bioRxiv preprint doi: https://doi.org/10.1101/2024.05.08.593125; this version posted May 8, 2024. The copyright holder for this preprint (which was not certified by peer review) is the author/funder. This article is a US Government work. It is not subject to copyright under 17 USC 105 and is also made available for use under a CC0 license.

886 **Supplemental figure 2.**

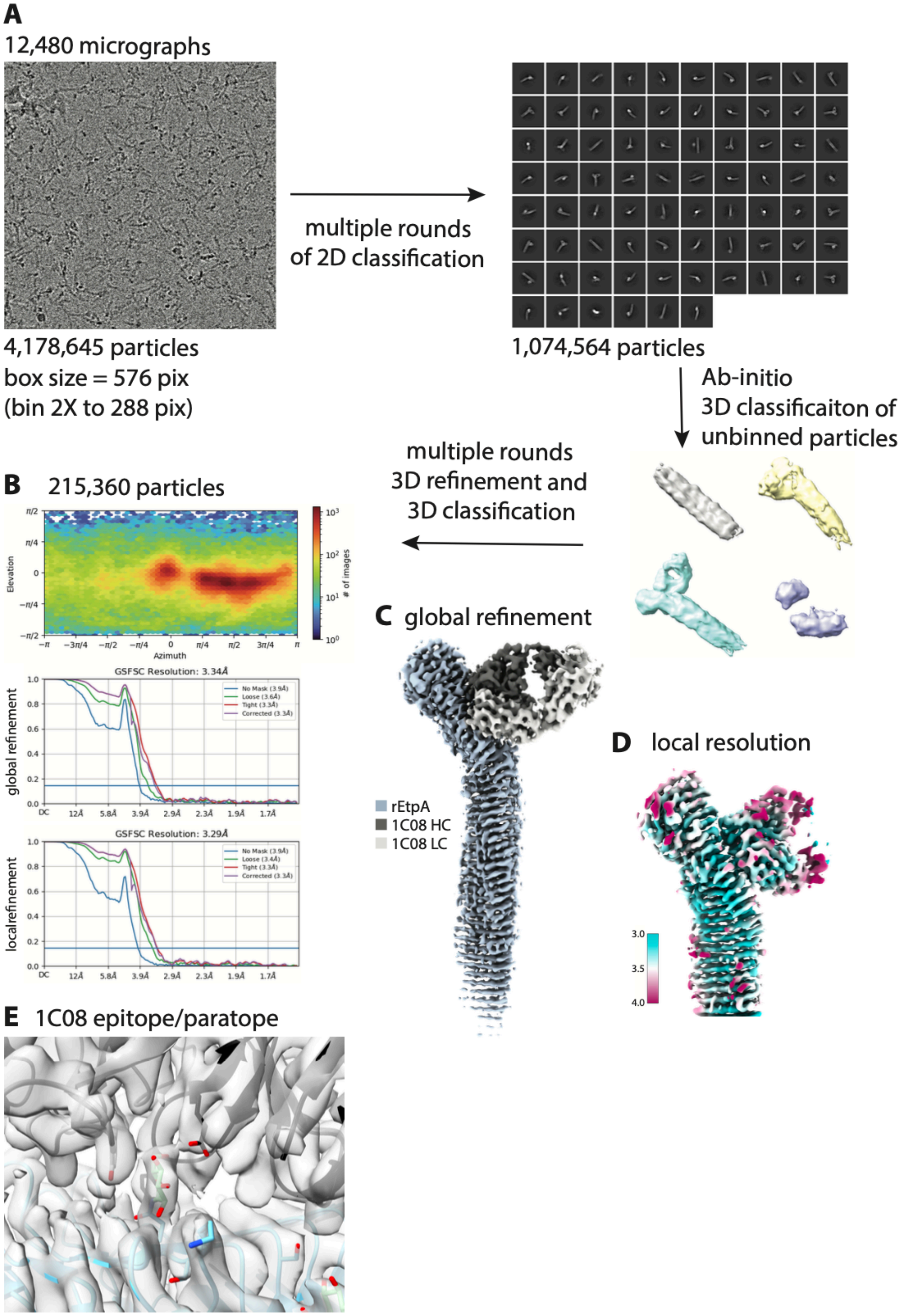

887

bioRxiv preprint doi: https://doi.org/10.1101/2024.05.08.593125; this version posted May 8, 2024. The copyright holder for this preprint (which was not certified by peer review) is the author/funder. This article is a US Government work. It is not subject to copyright under 17 USC 105 and is also made available for use under a CC0 license.

Supplemental figure 2. Cryo-EM data processing workflow for rEtpA-1C08 complex

Simplified cryo-EM data processing workflow including **A** representative example of an aligned and dose-weighted micrograph (lowpass filtered to 5Å), 2D and 3D class averages, and particle counts at each step. **B.** Angular distribution and Fourier shell correlation plots for the final 3D reconstruction along with the final particle count. **C.** Sharpened map colored by domain. **D.** Map colored by local resolution estimate. **E.** View of the map-model fit in the epitope/paratope region.

bioRxiv preprint doi: https://doi.org/10.1101/2024.05.08.593125; this version posted May 8, 2024. The copyright holder for this preprint (which was not certified by peer review) is the author/funder. This article is a US Government work. It is not subject to copyright under 17 USC 105 and is also made available for use under a CC0 license.

Supplemental figure 3.

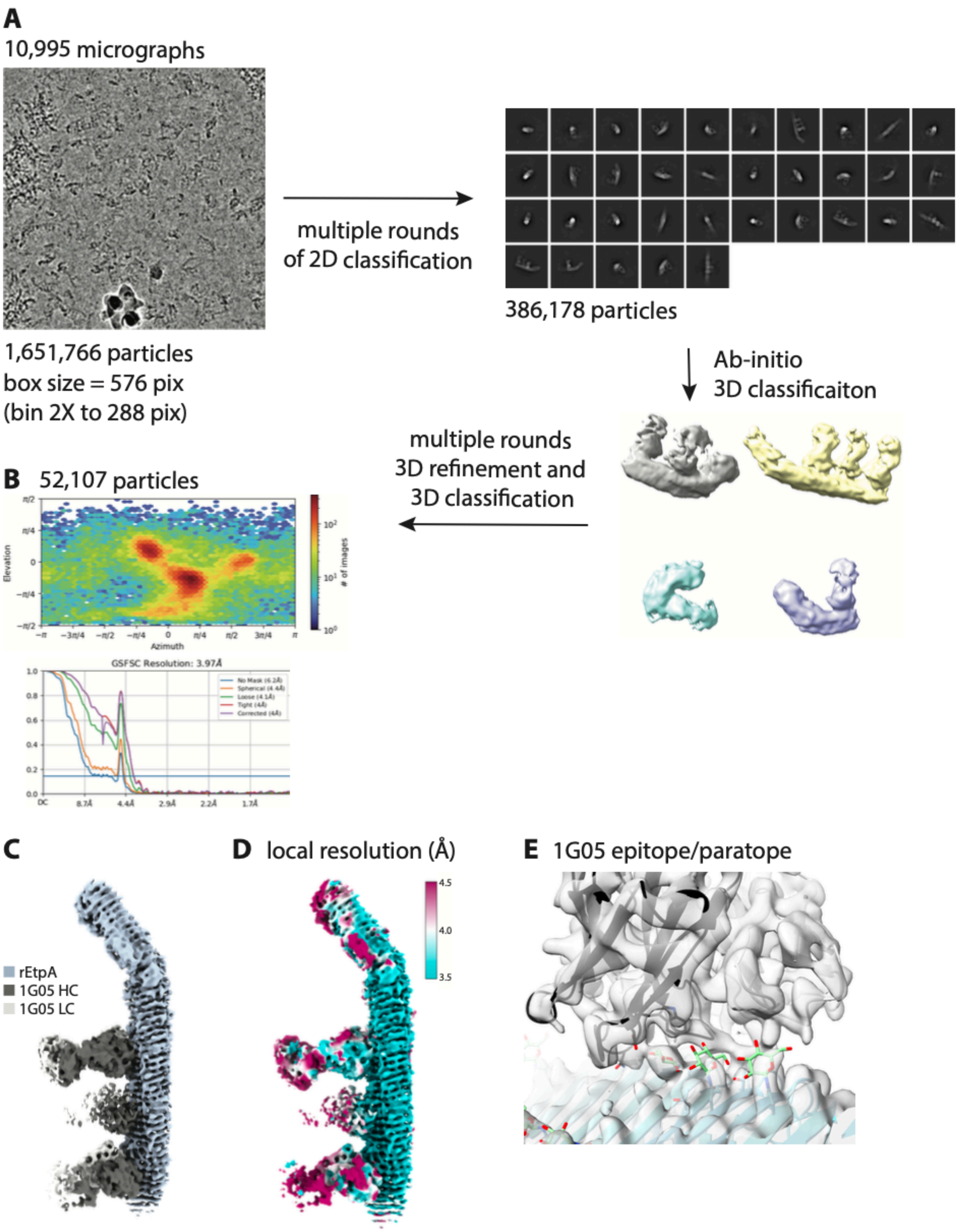

bioRxiv preprint doi: https://doi.org/10.1101/2024.05.08.593125; this version posted May 8, 2024. The copyright holder for this preprint (which was not certified by peer review) is the author/funder. This article is a US Government work. It is not subject to copyright under 17 USC 105 and is also made available for use under a CC0 license.

Supplemental figure 3. Cryo-EM data analysis for rEtpA-1G05 complex

Simplified cryo-EM data processing workflow including representative (**A**) aligned and dose-weighted micrograph (lowpass filtered to 5Å), 2D and 3D class averages, and particle counts at each step. **B.** Angular distribution and Fourier shell correlation plots for the final 3D reconstruction along with the final particle count. **C.** Sharpened map colored by domain. **D.** Map colored by local resolution estimate. **E.** View of the map-model fit in the epitope/paratope region.

bioRxiv preprint doi: https://doi.org/10.1101/2024.05.08.593125; this version posted May 8, 2024. The copyright holder for this preprint (which was not certified by peer review) is the author/funder. This article is a US Government work. It is not subject to copyright under 17 USC 105 and is also made available for use under a CC0 license.

Supplemental figure 4

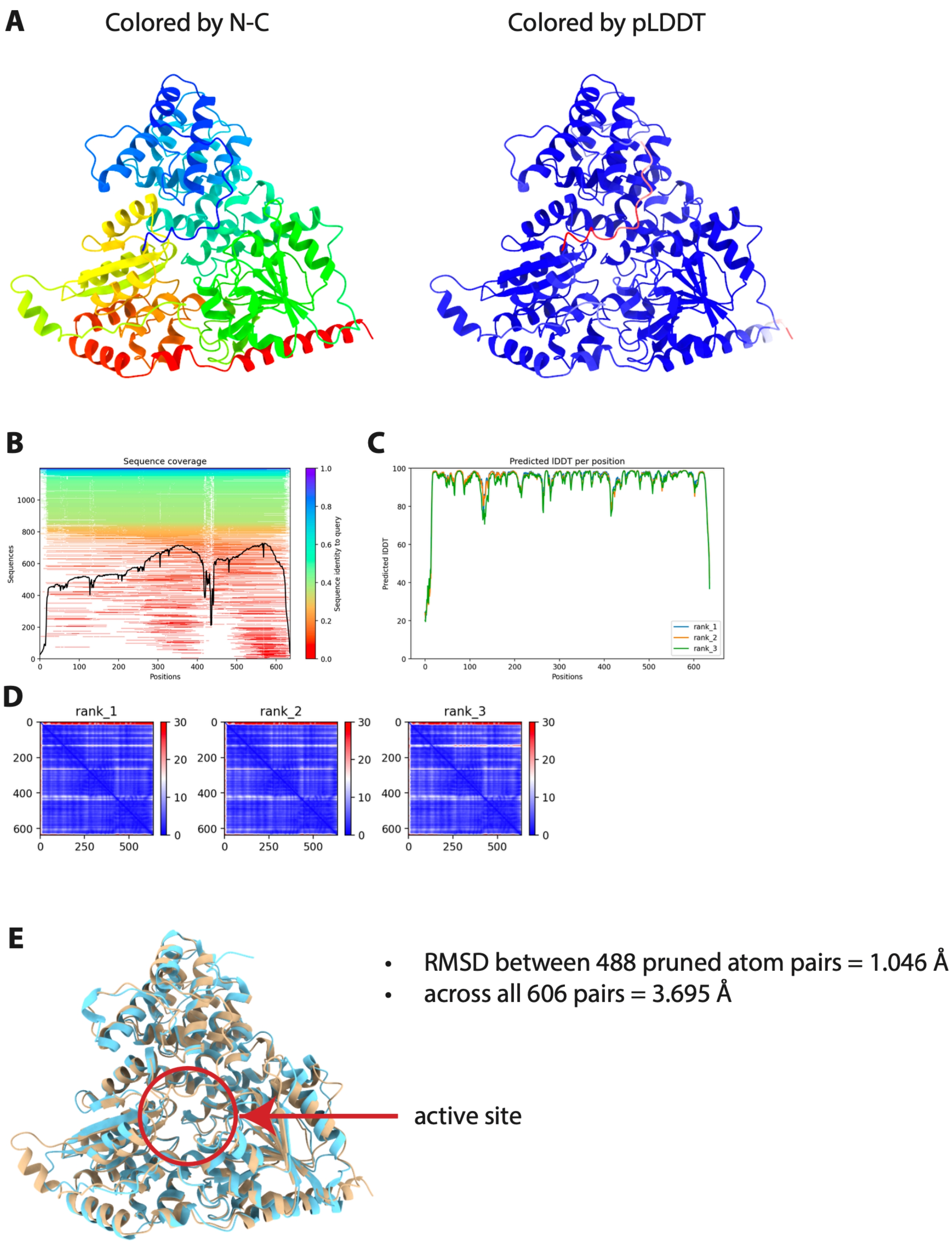

bioRxiv preprint doi: https://doi.org/10.1101/2024.05.08.593125; this version posted May 8, 2024. The copyright holder for this preprint (which was not certified by peer review) is the author/funder. This article is a US Government work. It is not subject to copyright under 17 USC 105 and is also made available for use under a CC0 license.

Supplemental figure 4. AlphaFold2 prediction of EtpC structure and structure-based alignment with the HMW1C crystal structure.

A. Predicted structure of EtpC colored with a rainbow color mapping from the N-terminus to C-terminus and **(B)** by prediction LDDT confidence store. **C.** Multiple sequence alignment coverage. **D.** Predicted LDDT score by residue position. **E.** Predicted alignment error matricies. **F.** Structure-based alignment of predicted EtpC structure with the crystal structure of HMW1C (PDBID:3Q3E).

bioRxiv preprint doi: https://doi.org/10.1101/2024.05.08.593125; this version posted May 8, 2024. The copyright holder for this preprint (which was not certified by peer review) is the author/funder. This article is a US Government work. It is not subject to copyright under 17 USC 105 and is also made available for use under a CC0 license.

918

919 **Supplemental figure 5.**

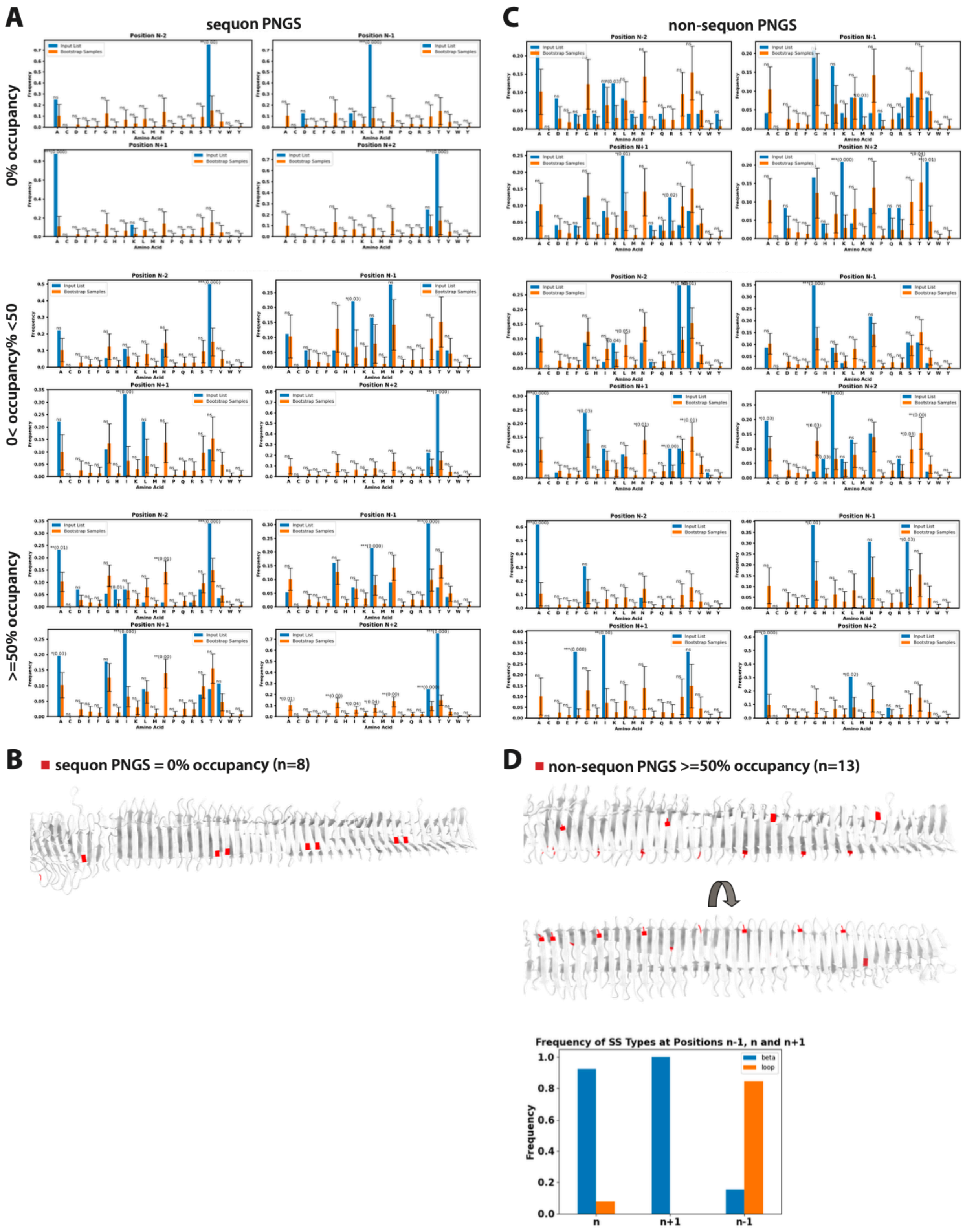

920

bioRxiv preprint doi: https://doi.org/10.1101/2024.05.08.593125; this version posted May 8, 2024. The copyright holder for this preprint (which was not certified by peer review) is the author/funder. This article is a US Government work. It is not subject to copyright under 17 USC 105 and is also made available for use under a CC0 license.

Supplemental figure 5. Statistical analysis of residues flanking PNGS

Statistical analysis of amino acid type frequencies at the 4 sites immediately upstream and downstream of each canonical sequon PNGS broken down by occupancy percentage as determined by mass-spectrometry. Blue bars are the average frequencies for the input residue list and orange bars are average frequencies for 1000 permutation samples with error bars and statistical significance measurements broken down by minor significance (*), median significance (**), and high significance (***) along with corresponding p-values. **B.** rEtpA structure with the 8 sequon PNGS sites with 0% occupancy colored red. **C.** Same as in A but for all non-canonical sequon PNGS. **D.** rEtpA structure showing all non-sequon PNGS with ≥ 50% occupancy colored red along with a bar plot showing the frequency of secondary structure types at the PNGS and the two residues immediately upstream and downstream of the site.

bioRxiv preprint doi: https://doi.org/10.1101/2024.05.08.593125; this version posted May 8, 2024. The copyright holder for this preprint (which was not certified by peer review) is the author/funder. This article is a US Government work. It is not subject to copyright under 17 USC 105 and is also made available for use under a CC0 license.

Supplemental figure 6

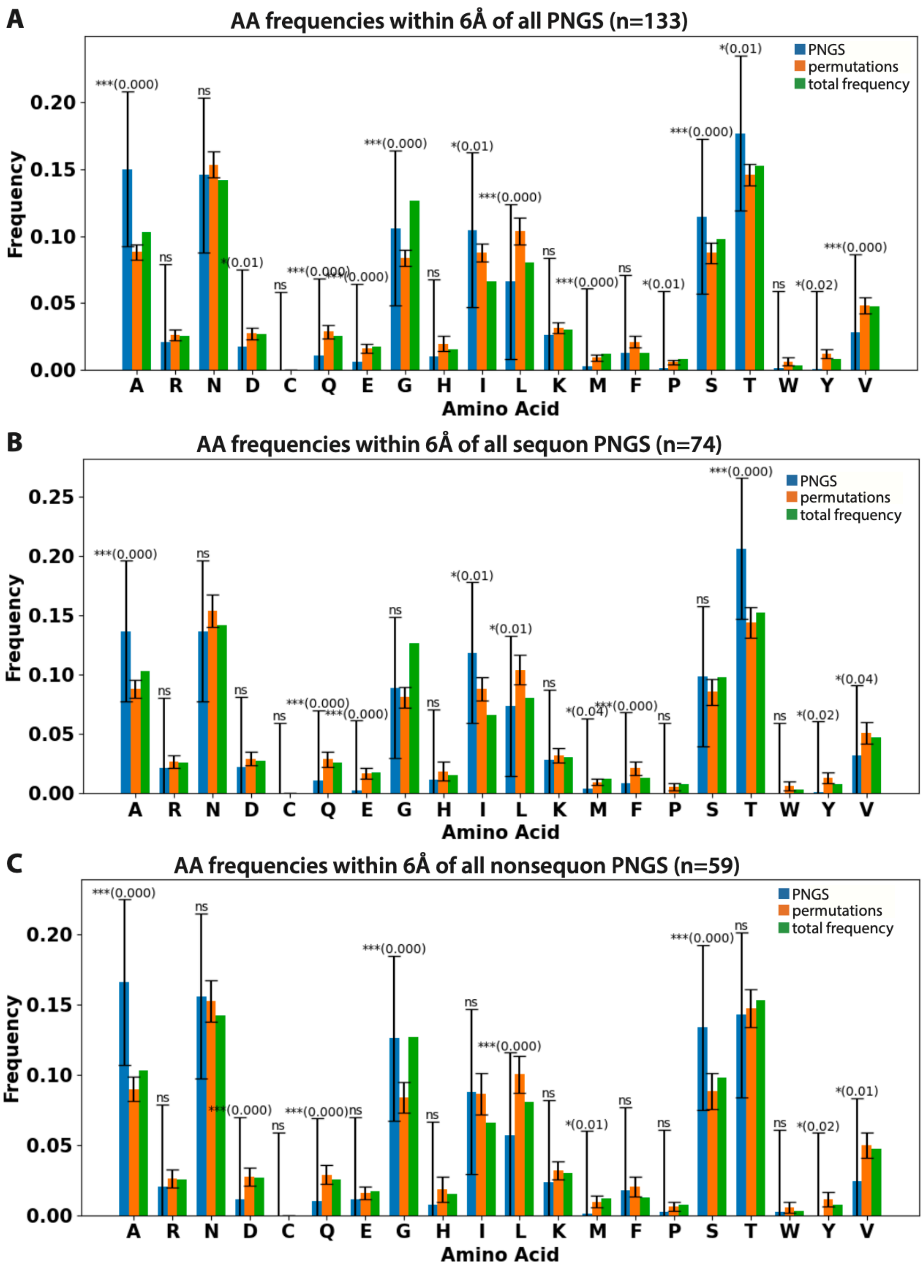

Supplemental figure 6. Statistical analysis of local structural environment around PNGS

bioRxiv preprint doi: https://doi.org/10.1101/2024.05.08.593125; this version posted May 8, 2024. The copyright holder for this preprint (which was not certified by peer review) is the author/funder. This article is a US Government work. It is not subject to copyright under 17 USC 105 and is also made available for use under a CC0 license.

A. Bar plot showing the average frequency of each amino acid type within 6Å of all PNGS, canonical sequon PNGS (**B**), and non-canonical sequon PNGS (**C**). Blue bars are frequencies for the input list of residues, orange bars are the average frequencies across all 1000 permutation samples (with error bars and significance measures as described in Supplemental figure 5), and green bars are the frequency of that amino acid within rEtpA.

bioRxiv preprint doi: https://doi.org/10.1101/2024.05.08.593125; this version posted May 8, 2024. The copyright holder for this preprint (which was not certified by peer review) is the author/funder. This article is a US Government work. It is not subject to copyright under 17 USC 105 and is also made available for use under a CC0 license.

Supplemental Figure 7

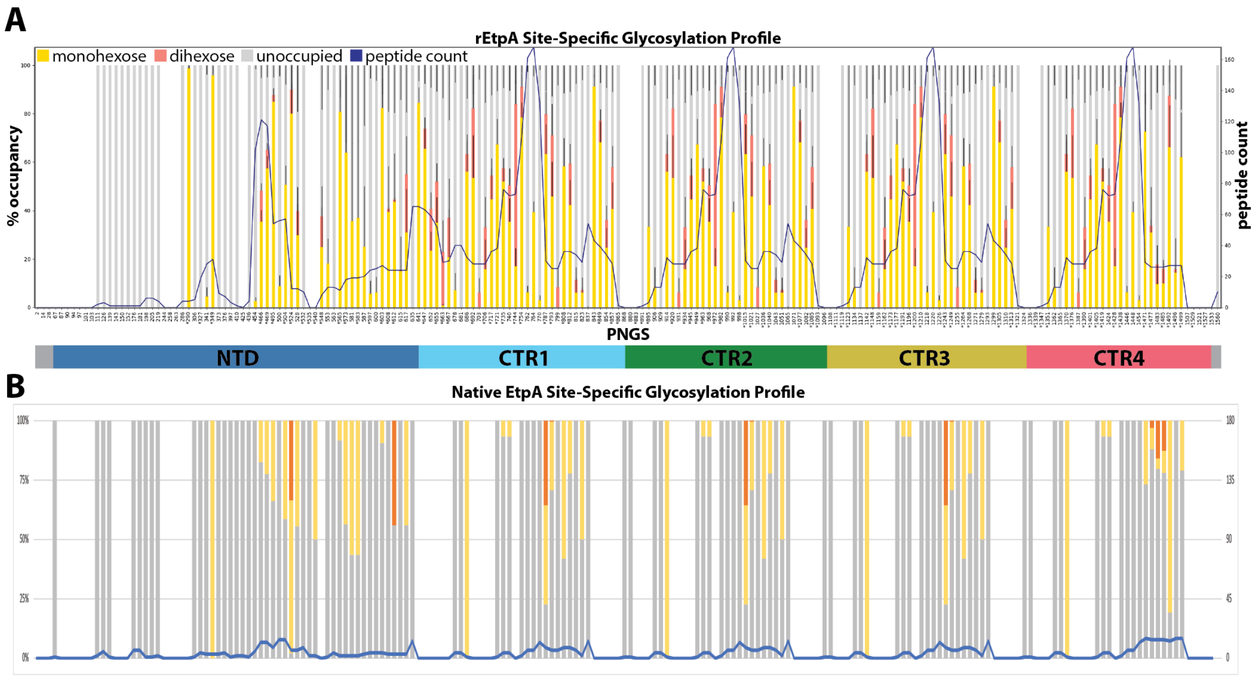

Supplemental figure 7. Site-specific glycosylation analysis of native EtpA.

A. Glycosylation profile for rEtpA reproduced from Figure 3. **B**. Glycosylation profile of native EtpA from ETEC strain H10407. Left axis is % occupancy and right axis is peptide count. Note the much lower peptide counts for native EtpA.

bioRxiv preprint doi: https://doi.org/10.1101/2024.05.08.593125; this version posted May 8, 2024. The copyright holder for this preprint (which was not certified by peer review) is the author/funder. This article is a US Government work. It is not subject to copyright under 17 USC 105 and is also made available for use under a CC0 license.

Supplemental figure 8

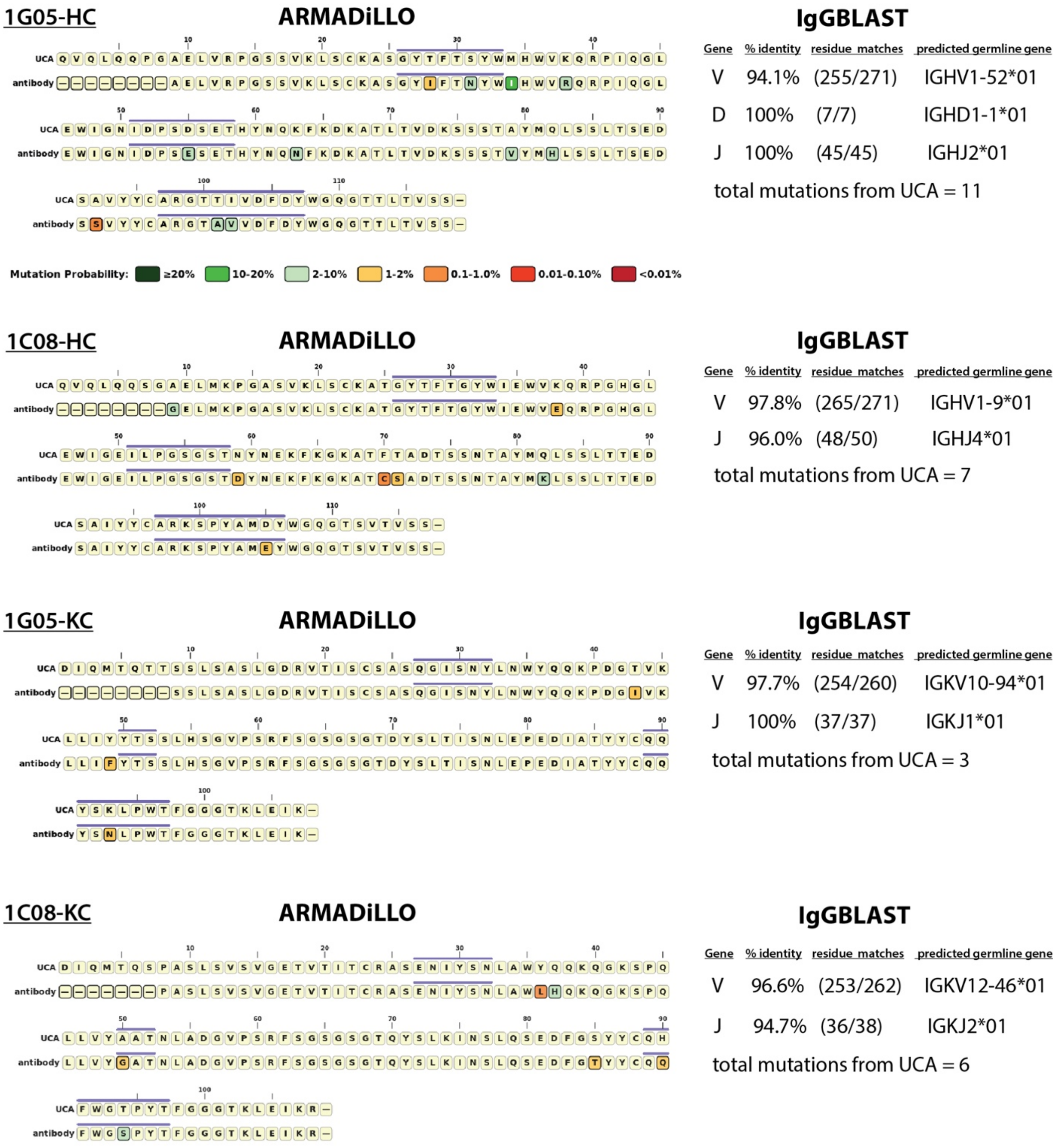

Supplemental figure 8. Germline gene IgGBlast and somatic hypermutation analysis by ARMADILLO. IgGBlast results summaries for 1C08 and 1G05 heavy chains (HC) and kappa chains (KC) showing the top match for V, D, and J mouse germline genes. Next to the

bioRxiv preprint doi: https://doi.org/10.1101/2024.05.08.593125; this version posted May 8, 2024. The copyright holder for this preprint (which was not certified by peer review) is the author/funder. This article is a US Government work. It is not subject to copyright under 17 USC 105 and is also made available for use under a CC0 license.

summary tables are the percent identity to the top matching genes along with the gene names and the total number of somatic hypermutations (SHM) away from the predicted unmutated common ancestor (UCA). Also shown are output plots from the program ARMADILLO with SHM sites shown and scored by their probability, with red being the least probable and green being the most probable.

bioRxiv preprint doi: https://doi.org/10.1101/2024.05.08.593125; this version posted May 8, 2024. The copyright holder for this preprint (which was not certified by peer review) is the author/funder. This article is a US Government work. It is not subject to copyright under 17 USC 105 and is also made available for use under a CC0 license.

Supplemental figure 9

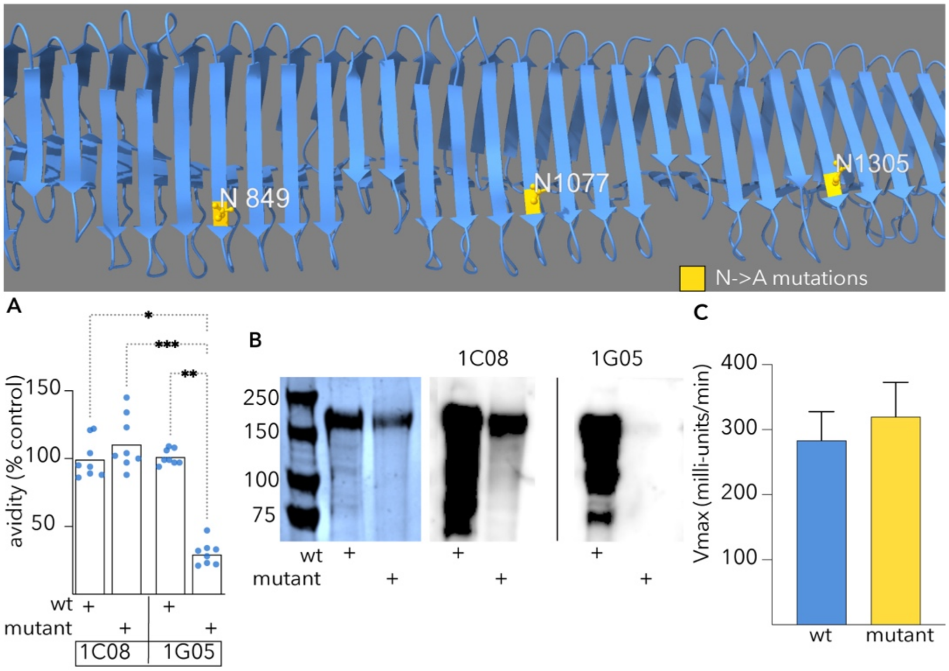

Supplemental figure 9. Mutation of 1G05 epitope glycan sites impacts avidity

Figure at top depicts relative location of asparagine (N) to alanine (A) mutations to the putative 1G05 epitope. **A.** mAb 1G05 exhibits decreased avidity for recombinant mutant EtpA with N to A substitutions within C-terminal repeat region at positions 849, 1077, and 1305. Avidity indices (AI) were determined by kinetic ELISA with and without addition of 8 M urea as the chaotropic agent. AI (%) = (Vmax with urea)/(Vmax without urea) and expressed as % of the wild type recombinant protein. Comparisons by Kruskal-Wallis (n=8 technical replicates/group from 2 independent experiments) ***=0.0003, **=0.0037, *=0.03. **B.** Immunoblot recognition of wild type and mutant protein by 1C08 and 1G05. PAGE image (left) indicates protein loading and MW markers. **C.** Blood group A binding by wild type and mutant protein in kinetic ELISA assay.

bioRxiv preprint doi: https://doi.org/10.1101/2024.05.08.593125; this version posted May 8, 2024. The copyright holder for this preprint (which was not certified by peer review) is the author/funder. This article is a US Government work. It is not subject to copyright under 17 USC 105 and is also made available for use under a CC0 license.

Supplemental figure 10

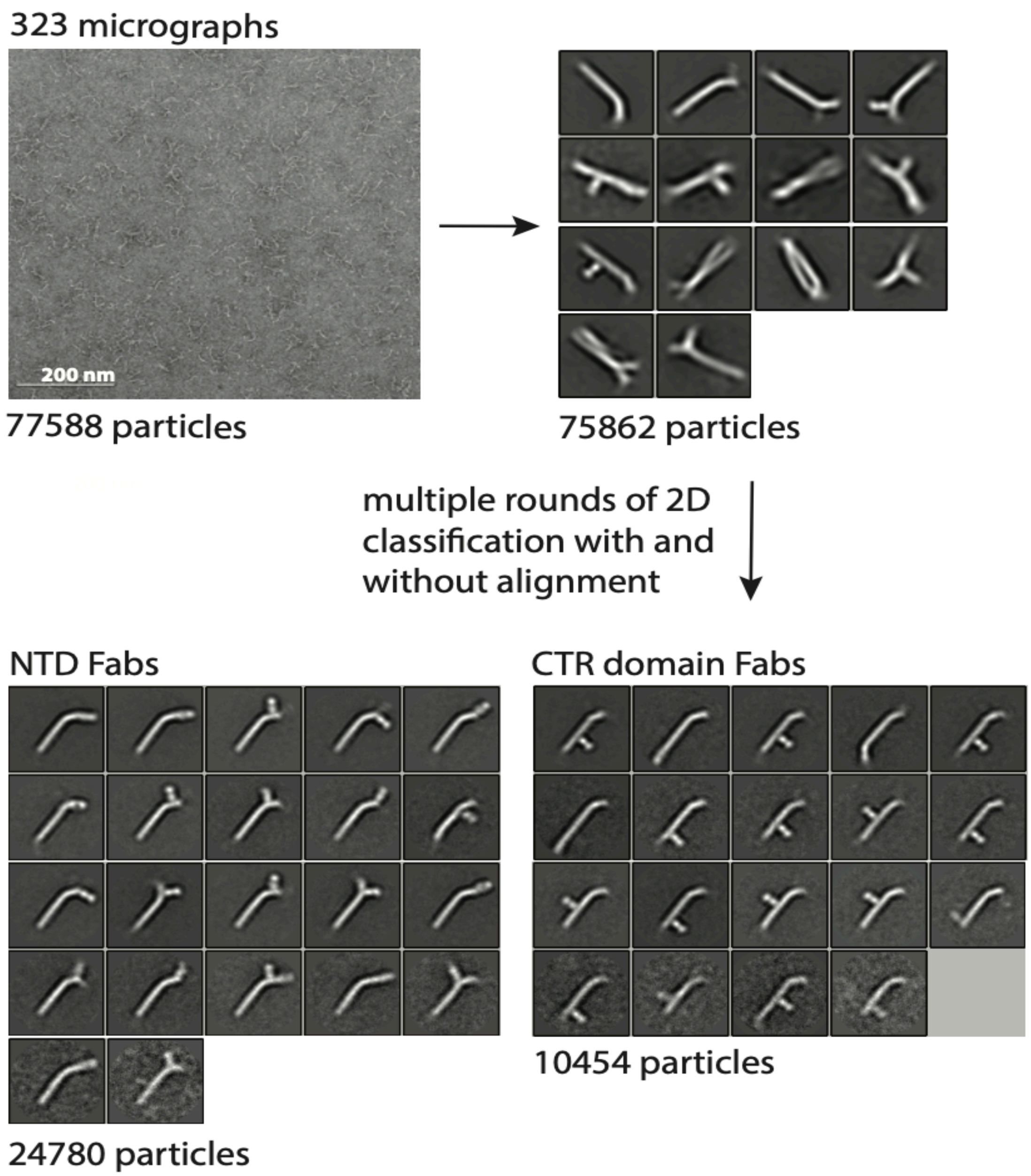

Supplemental figure 10. EMPEM processing workflow

Simplified negative stain EMPEM data processing workflow including a representative micrograph, 2D class averages, and particle counts at each step.

bioRxiv preprint doi: https://doi.org/10.1101/2024.05.08.593125; this version posted May 8, 2024. The copyright holder for this preprint (which was not certified by peer review) is the author/funder. This article is a US Government work. It is not subject to copyright under 17 USC 105 and is also made available for use under a CC0 license.

Supplemental figure 11

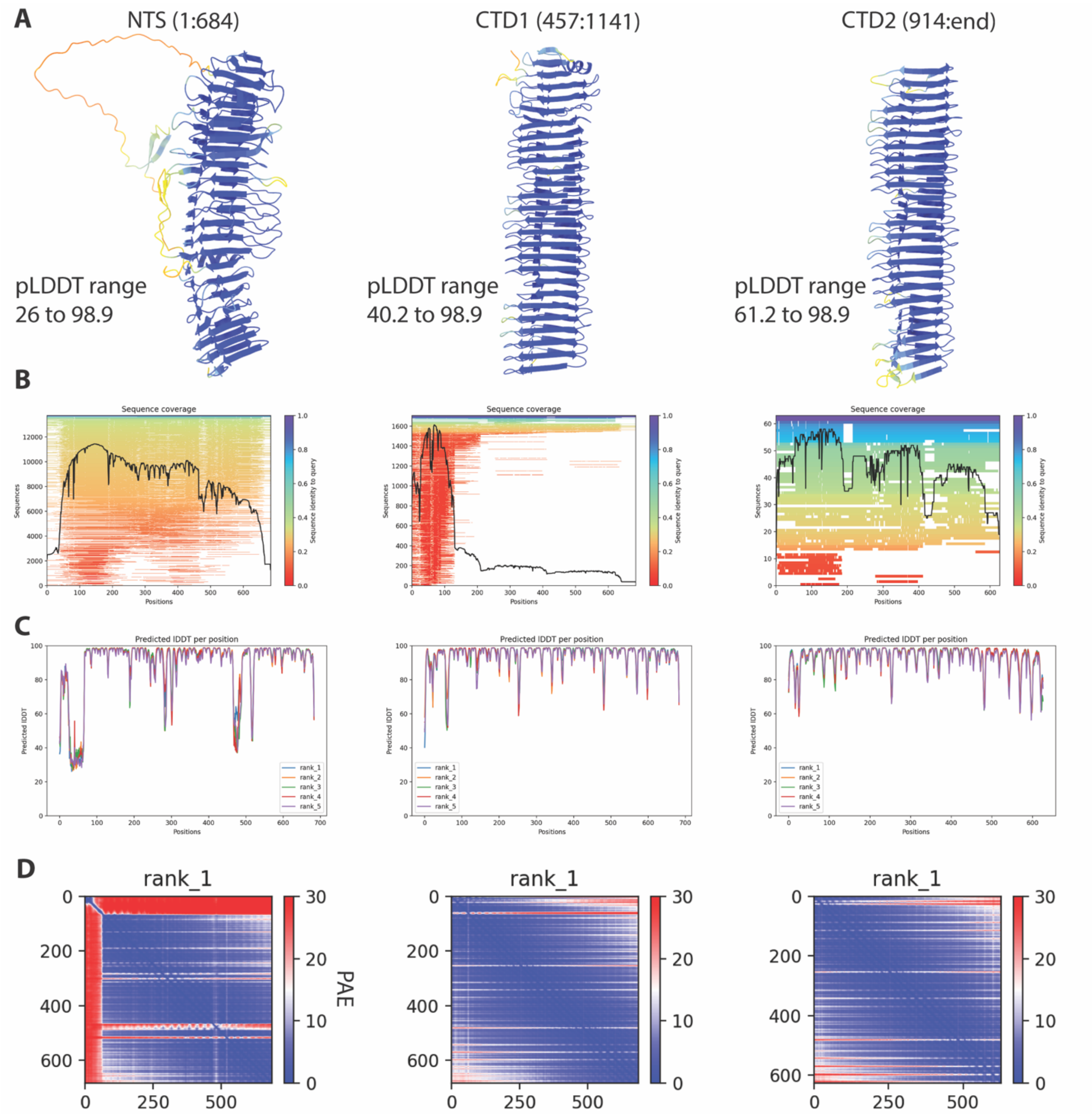

Supplemental figure 11. AlphaFold2 predictions for full-length EtpA

A. AlphaFold2 top ranking model colored by pLDDT score (blue = high). **B**. Per-residue sequence coverage and identity. **C**. Per-residue pLDDT scores. **D**. PAE (predicted aligned error) matrix.

bioRxiv preprint doi: https://doi.org/10.1101/2024.05.08.593125; this version posted May 8, 2024. The copyright holder for this preprint (which was not certified by peer review) is the author/funder. This article is a US Government work. It is not subject to copyright under 17 USC 105 and is also made available for use under a CC0 license.

Supplemental tables

Supplemental table 1

supplemental table 1 strains and plasmids			
strain	description/genotype	reference	
Top10	F-mcrA (mrr-hsdRMS-mcrBC) 80lacZM15 lacX74 recA1 araD139 (ara-leu)7697 galU galK – rpsL(StrR) endA1 nupG	Invitrogen	
jf1696	Top10(pJL017/pJL030), AmpR, CmR	[48,71]	
jf2826	LMG194 *fliC*::KmR	[48]	
jf3013	Top10(pQL211/pJL030), AmpR, CmR	this study	
jf3099	H10407 *fliC*::KmR	[48]	
jf5090	Top10(pMH4/pJL030) AmpR, CmR	this study	
jf5381	jf2826(pJL030)		
jf5500	jf5381(pJL030/pTV005) AmpR, CmR	this study	
H10407	wild type ETEC strain *etpBAC* serotype O78:H11, LT/STh/STp	[17,78]	
plasmid	description	reference(s)	addgene number
pJL030	pACYC184 based etpC expression plasmid CmR	[48,123]	53533
pJL017	etpBA cloned into pBAD/Myc- His A*, with etpA in-frame with myc and 6His coding regions	[35]	53532
pJMF1028	linker scanning mutant of pJL017 with transprimer insertion at 3258 of *etpA*	[48]	
pMH4	EtpA-amplified from pJMF1028, encodes EtpA (AA 1-1086)	this study	
pQL211	N-terminal region EtpA subclone (AA 1-607) generated with primers jf02214.3/jf022114.4 cloned in-frame with myc and 6His coding regions of pBAD/Myc- His A.	[124]	
pTV005	Mutant EtpA expression plasmid bearing N849A, N1077A, and N1305A mutations	this study	
KmR=kanamycin resistant; AmpR=beta-lactamase/ampicillin resistant; CmR=chloramphenicol resistant.			

Supplemental table 2

supplemental table 2: primers	
primer	sequence 5'->3'
jf051716.1	GAGGAATTAACC**ATGG**TGGTGAAATTCATGTCAG
jf082718.1	TTGTTCTAGAAAGC**TT**CCCAACTTTATTGTGTTTAAACA
jf022014.3	ACTTCAAACCGTGTGAATATATCTATTAATAAGCTTGGGCCCGAAC
jf022014.4	GTTCGGGCCCAAGCTTATTAATAGATATATTCACACGGTTTGAAGT
pBAD reverse	GATTTAATCTGTATCAGG
Regions in bold correspond to 5' end of *etpB* sequence for jf051716.1, and to 3' end of the GPS4 Tn7L transprimer sequence for jf082718.1. Underlined nucleotides represent *NcoI* and *HindIII* sites, respectively.	

bioRxiv preprint doi: https://doi.org/10.1101/2024.05.08.593125; this version posted May 8, 2024. The copyright holder for this preprint (which was not certified by peer review) is the author/funder. This article is a US Government work. It is not subject to copyright under 17 USC 105 and is also made available for use under a CC0 license.

Supplemental table 3

Cryo-EM data collection, refinement, and validation statistics

	rEtpA:1C08 Fab<br>(EMDB-xxxx)<br>(PDB xxxx)	rEtpA:1G05 Fab<br>(EMDB-xxxx)<br>(PDB xxxx)
**Data collection and processing**		
Magnification	97,0000	97,0000
Voltage (kV)	200	200
Electron exposure (e–/Å^2)	47	47
Defocus range (μm)	~-0.25:-2.0um	~-0.4:-2.0um
Pixel size (Å)	0.725Å	0.725Å
Symmetry imposed	none	none
Initial particle images (no.)	4,178,645	1,651,766
Final particle images (no.)	215,360	52,107
Map resolution (Å)<br>FSC threshold	3.34Å<br>0.143	3.97Å<br>0.143
Map resolution range (Å)	~3-5Å	~3.5-5Å
Map sharpening B factor (Å^2)	110.5	380.4
**Refinement**		
**Initial model used (PDB code)**	**AlphaFold2 and SABPred predictions**	**AlphaFold2 and SABPred predictions**
Model composition<br>Non-hydrogen atoms<br>Protein residues<br>Ligands	 9416<br>1260<br>39 BGC	 9444<br>1260<br>38 BGC
R.m.s. deviations<br>Bond lengths (Å)<br>Bond angles (°)	 0.011<br>1.318	 0.01<br>1.03
Validation<br>MolProbity score<br>Clashscore<br>Poor rotamers (%)<br>CaBLAM outliers (%)	 2.03<br>14.87<br>0.51<br>2.8	 2.02<br>15.05<br>0.41<br>3.53
Ramachandran plot<br>Favored (%)<br>Allowed (%)<br>Disallowed (%)	 94.90<br>4.63<br>0.48	 95.14<br>4.63<br>0.24
EMRinger score	3.6	2.7
